# Strategic Realignment in Healthcare Simulation Education: Shifting Focus From Assistants to Technician Training Pathways

**DOI:** 10.7759/cureus.58746

**Published:** 2024-04-22

**Authors:** Refka Al-Bayati, Julia Micallef, Krystina M Clarke, Samyah Siraj, Adam Dubrowski

**Affiliations:** 1 Health Sciences, Ontario Tech University, Oshawa, CAN; 2 maxSIMhealth Research, Ontario Tech University, Oshawa, CAN

**Keywords:** competency-based teaching, competency framework, integrated knowledge translation (ikt), undergraduate curriculum, healthcare simulation, simulation education

## Abstract

Simulation is vital for healthcare training, yet workforce challenges persist. This article details the development of an undergraduate minor program to address these issues and enhance simulation education. Initially conceived for simulation assistants, the program shifted focus to training simulation technicians. Informed by industry insights, the curriculum aligns with accreditation standards, emphasizing practical knowledge. Integrated knowledge translation (iKT) fosters collaboration, ensuring program relevance. Stakeholder feedback guided program refinement, addressing concerns of role delineation and alignment with certification frameworks. The program's evolution involved enhancing competency frameworks, validation through surveys, and forming partnerships for practical training. A certification committee ensures ongoing alignment with industry standards. This collaborative effort aims to produce graduates prepared for the dynamic field of healthcare simulation technology, thereby improving patient outcomes and advancing simulation education.

## Editorial

Simulation in healthcare is indispensable for replicating real-life scenarios, empowering professionals to practice, learn, and prepare for genuine situations in a secure environment. It also plays a critical role in safety testing and quality improvement, allowing practitioners to simulate real-world scenarios and enhance patient safety through immersive experiences. Successful simulation implementation relies on integrating authentic cases, simulation-specific curriculum, technology, and effective management. Currently, simulation facilities, often called simulation laboratories or centers, are managed by skilled simulation technicians, typically with a healthcare background [1].

The increasing complexity of digital technologies in the simulation market has prompted healthcare professionals, such as nurses and paramedics, to transition from their primary roles to simulation technicians. This shift is driven by the growing intricacy of digital tools, which demand specialized expertise for effective utilization. This adds pressure to an already stretched healthcare system, further straining its limited resources [1]. To tackle this issue, we have initiated the development of a minor program within the Faculty of Health Sciences at Ontario Tech University, providing a direct pathway from undergraduate studies to simulation roles [2]. This novel program aims to equip graduates with the necessary skills and knowledge to enter the field and address the existing gap in the simulation workforce. Our program's development took a different path initially, as we created a minor program designed to train “simulation assistants” to support simulation technicians in their daily tasks [2]. However, through a process of collaborative discovery, a process where individuals or groups work together to explore, investigate, and uncover new information, insights, or solutions to a problem we identified that our initial approach was not optimal. Consequently, we focused on establishing a program for preparing undergraduate Bachelor of Health Science (BHSc) students to be simulation technicians instead. This editorial aims to highlight the critical role of such a collaborative discovery approach and nests it in an integrated knowledge translation (iKT) framework [3].

From the onset, our program research and development strategy embraced the tenets of iKT to fortify the pertinence and efficacy of our program. By nurturing partnerships between researchers and knowledge users (KUs), encompassing healthcare practitioners and educators, iKT cultivates a reciprocal learning dynamic throughout the research and development journey [3]. This collaborative ethos enables us to tailor our curriculum to meet the precise needs of the healthcare sector, ensuring that graduates emerge equipped with practical acumen directly relevant to real-world scenarios. Through sustained dialogue and cooperation with stakeholders, we aimed to co-create a curriculum that yields mutual benefits, fostering a participatory process. By embracing the principles of iKT, we aimed to harbor confidence that our program will not only confront present workforce dilemmas but also spearhead advancements in simulation education, ultimately enhancing patient outcomes across healthcare domains.

Initial solution

Our initial strategy focused on introducing the "simulation assistant" position to tackle the challenge of healthcare workers shifting to simulation roles [2]. This new role sought to engage health science students in bridging a workforce gap by introducing a position that complements that of a simulation technician. Suggested responsibilities included tasks such as maintaining the cleanliness of manikins and setting up simulation scenarios. Our plan included establishing a competency-based minor program within the BHSc at Ontario Tech University. To begin, we conducted a comprehensive literature review, utilizing previous frameworks [4], exam blueprints from various accreditation bodies (http://surl.li/lnjqp), and artificial intelligence in the form of ChatGPT 3.5. This process helped us develop an extensive initial competency framework, which was to be refined into suitable competencies for simulation assistants during a focus group session scheduled during an annual meeting hosted by Simulation Canada (SIM EXPO 2023 in Ottawa, Canada).

During this one-hour session, nine experts in the field with diverse backgrounds, including nursing, paramedicine, and physiotherapy, spanning academia, healthcare delivery, and health sales, were in attendance. We presented our framework and introduced the concept of a new role within simulation teams: the simulation assistant. In the original conception, this role aimed at enhancing the capabilities of simulation technicians through additional support. However, this concept faced swift opposition and received constructive feedback from a subset of our group, largely stemming from concerns regarding compensation and the notion that individuals with minimal training, such as volunteer high school students, could effectively undertake the responsibilities of an assistant. The prevailing notion suggested that any volunteer high school or undergraduate student could serve as a simulation assistant, potentially resulting in graduates from the minor program receiving lower pay compared to counterparts with bachelor's degrees in equivalent roles. Another point highlighted by the group pertained to concerns surrounding the delineation of roles and responsibilities. Some apprehensions emerged regarding potential overlap between tasks assigned to simulation assistants and those typically handled by technicians. The group felt that this ambiguity would further add to the current confusion regarding the taxonomy used to describe support personnel participating in simulation, such as simulation technicians, technologists, operators, and simulationists.

Consequently, the group emphasized the importance of aligning the new training program with existing certification frameworks. They recommended adopting the most current terminology and job designations, while structuring the curriculum to ensure that graduates are equipped with the necessary skills to enter the workforce seamlessly, in line with industry standards. Instead of solely focusing on introducing a new professional designation for simulation assistants, the group suggested prioritizing the alignment of the training program with established certification standards, such as the Certified Healthcare Simulation Operations Specialist (CHSOS) certification provided by the Society for Simulation in Healthcare (SSH). This shift in focus underscores the need to concentrate on designing education pathways that produce graduates who meet industry requirements, rather than solely emphasizing differentiation in job titles.

Strategic pivot

Consequently, we underwent a significant pivot in our strategy. Instead of pursuing the introduction of simulation assistants, we shifted our focus towards enhancing the educational pathway for simulation technicians, aligning our curriculum closely with established accreditation standards such as the SSH. 

This strategic shift not only served to elevate the status of our graduates but also impacted the research and development process. First, we revamped our competency framework using the Six-Step Model for Developing Competency Frameworks in the Healthcare Professions developed by Batt et al. [5] to align with the role of a simulation technician. This involved going back to the "drawing board" and refining our framework, which would constitute the first two steps of the model: identifying purpose, intended uses, scope, stakeholders, and theoretically informed ways of identifying the contexts of complex, "real-world" professional practice. Additionally, we devised a survey to validate the robustness of the framework, which would fulfill the third and fourth steps: ​​aligned methods and means by which practice can be explored and the identification and specification of competencies required for professional practice. Next, we initiated the search for partners who could facilitate a practicum component of the minor program at their sites, addressing the highlighted importance of students comprehending the contextual nuances of their roles as simulation technicians within real-world settings. We are also expanding our stakeholder group to include key individuals who worked and maintained the CHSOS certification, as well as members of other key groups, such as SimGHOSTS who aim to shape the landscape of personnel supporting simulation activities. For instance, to align with our commitment to involving industry experts in our program development, we have initiated one-hour meetings with a select group of professionals highly engaged in SSH and intimately familiar with the examination blueprints utilized by accreditation bodies. These structured sessions serve as a platform for us to discern key priorities within these blueprints and tailor our curriculum accordingly, emphasizing practical knowledge over mere theory. We engage experts in discussions regarding the critical components of the examinations, strategies for seamless integration into our curriculum, and avenues for preparing students to undertake these examinations immediately upon graduation. Furthermore, we are currently establishing our certification committee, composed of a carefully selected group of experts with whom we have collaborated. This committee will play a pivotal role in guiding us through every stage of program development, ensuring alignment with industry standards, and holding us accountable if we stray from our intended course. Additionally, we have constructed a survey utilizing our developed competency framework, which we will soon launch to assess the practical applicability of these competencies in real-world scenarios, juxtaposed against their significance in the certification exams administered by SSH. 

Conclusion

In conclusion, our journey in developing a minor program for simulation technicians at Ontario Tech University has been challenging and enlightening. Simulation in healthcare is undeniably crucial for preparing professionals and improving patient outcomes, but addressing workforce challenges and adapting to evolving industry needs require innovative approaches. Initially, our focus on introducing simulation assistants faced significant opposition and prompted a strategic pivot toward enhancing the educational pathway for simulation technicians. This shift was guided by insights from industry professionals and emphasized the importance of aligning our curriculum with accreditation standards while considering practical implications.

Our commitment to iKT, which is renewed by the critical pivot described here, ensures that our program remains relevant and effective by fostering collaboration between researchers and KUs, and by incorporating real-world experiences into our curriculum. Moving forward, our focus remains on collaboration, refinement, and validation, as we continue to engage with industry experts, establish partnerships, and refine our competency framework through surveys and consultations. By embracing feedback, recognizing contextual factors, and prioritizing practical knowledge, we aim to cultivate a program that not only addresses current workforce challenges but also contributes to the advancement of simulation education and ultimately enhances patient outcomes in healthcare. Through ongoing collaboration and innovation, we are confident in our ability to prepare graduates who are equipped to thrive in the dynamic and demanding field of healthcare simulation technology.
